# Diagnostic Value of Exhaled Carbon Monoxide as an Early Marker of Exacerbation in Children with Chronic Lung Diseases

**DOI:** 10.5402/2012/859873

**Published:** 2012-09-11

**Authors:** Karima A. Abd EL Khalek, Magda Y. EL Seify, Omneya I. Youssef, Mona M. Badr

**Affiliations:** Department of Pediatrics, Faculty of Medicine, Ain Shams University, Cairo 11321, Egypt

## Abstract

Chronic airways infection and inflammation are leading causes of morbidity and mortality in chronic lung diseases (CLD). Pulmonary exacerbations are major causes of morbidity in CLD. Exhaled carbon monoxide (eCO) is a product of endogenous metabolic processes whose presence in exhaled breath is considered an index of inflammatory processes. *Objective.* To evaluate carbon monoxide (eCO) as inflammatory marker for early detection of acute exacerbation in CLD. *Methods.* Case control study included 40 children with CLD (twenty in exacerbation, group I and twenty in quiescent period, group II) recruited from the Chest Clinic, Children's Hospital, Ain Shams University. Twenty apparently healthy children were included as controls (group III). *Results.* Patients' mean age was 9.98 ± 3.29 years: 24 (60%) males and 16 (40%) females. The mean eCO level among patients during exacerbation was 5.35 ± 1.35 (ppm) compared to 2.65 ± 0.49 (ppm) in quiescent stage and 1.30 ± 0.47 (ppm) in controls. eCO cutoff value discriminating cases and control was 1.5 (ppm) (sensitivity; 100% and specificity 70%) and cutoff value discriminating group I from group II was 3 (ppm) (sensitivity: 100% and specificity: 100%). *Conclusion.* Exhaled CO can be considered a noninvasive early marker of acute exacerbation of CLD.

## 1. Introduction

Chronic lung diseases (CLDs) including cystic fibrosis, asthma, chronic obstructive pulmonary disease (COPD), and other types of bronchiectasis represent a major challenge for health care [1]. Chronic infection and inflammation of the airways are the leading causes of morbidity and mortality in CLD [2]. Pulmonary exacerbations are a major cause of morbidity and decreased quality of life for patients with chronic lung diseases [3]. 

Exhaled breath analysis has enormous potential as an easy, non invasive means of monitoring inflammation and oxidative stress in the airway [4]. CO represents a product of endogenous metabolic processes whose presence on exhaled breath is considered as indices of inflammatory processes [5]. Exhaled Co not only can be used as an oxidative stress marker, but also can reflect the severity of chronic lung diseases (CLD). Also exhaled CO could serve as an indicator of acute exacerbations in children with CLD [6]. 

### 1.1. Aim of the Work

 The present study aimed to detect the diagnostic value of carbon monoxide (CO) as inflammatory marker for early detection of acute exacerbation of chronic lung diseases. 

## 2. Patients and Method

### 2.1. Study

Case control study was carried at the Chest Clinic, Children's hospital, Ain Sham University in the period from April 2010 to February 2011.

### 2.2. Subjects

The present study was carried out on forty (40) children with chronic lung diseases including bronchiectasis, interstitial lung disease, and cystic fibrosis their ages ranged from 5 to 17 years with a mean age 9.98 ± 3.29 years, twenty to four (60%) were males and sixteen (40%) were females. Twenty were in exacerbation (group I); they were 11 (55%) males and 9 (45%) females. Their ages ranged from 5 to 17 years with a mean age 9.98 ± 3.29 years. Criteria for pulmonary exacerbation were considered the presence of one or more of the following symptoms: increased cough, increased sputum production, change in the quality of the sputum (more purulent, increased thickness, or presence of blood), increased dyspnea, pleuritic chest pain, wheezing, increase of C-reactive protein (CRP), and worsening of FEV1% [7]. Twenty patients were not fulfilling these criteria and were considered to be in quiescent period (group II); they were 14 (70%) males and 6 (30%) females. Their ages ranged from 5 to 17 years with a mean age 10.58 ± 3.44 years. Another twenty healthy age- and sex- matched children without evidence of infection were included as a control group (group III), (50% males and 50% females with a mean age 7.65 ± 2.323 years). 

Informed consents were taken from parents or care givers of studied children. The local pediatric board ethically approved the study.

### 2.3. Exclusion Criteria

 Patients with diseases that may affect exhaled CO as bronchial asthma, tuberculosis, and diabetes mellitus were excluded from the study.

All patients were subjected to careful history taking, through clinical examination laying stress on chest examination; laboratory investigations: complete blood with total and differential picture using coulter counter (T660), erythrocyte sedimentation rate by Westergren method and C-reactive protein (CRP) using latex agglutination, radiological investigations including chest X-rays and CT scan and spirometric pulmonary function testing for selected cases.

**Exhaled Carbon Monoxide (CO) Measurement**

Exhaled CO levels were measured by portable breath CO monitor (Bedfont Scientific piCO+ Smokerlyzer (Pico+), UK). The Smokerlyzer measures breath CO levels in parts per million (ppm) based on the conversion of CO to CO_2_ over catalytically active electrode.

The subjects under the study were asked to inhale deeply and hold their breath for 15 seconds then they were asked to blow slowly into mouthpiece, aiming to empty lungs completely. The procedure was repeated two times, with 1 min of normal breathing between each time, and the mean value was used for analysis. During the procedure, the nose of the subjects was clamped. This was done to remove any contribution from the paranasal sinuses to the exhaled CO levels. The ppm and equivalent % carboxyhemoglobin (CoHb) level were measured and appeared on the display monitor. 

### 2.4. Statistical Analysis

Collected data were entered in a PC (personal computer) using SPSS program (statistical package for social science) version (16) to be analyzed as follows: quantitative variables as mean (SD) and range, qualitative variables as number and percentage. Chi-Square test X^2^, ANOVA, unpaired (Student's) *t*-test, and correlation coefficient test were performed. *P* values less than 0.05 were considered statistically significant. ROC curve was used to assess cut-off value, specificity, and sensitivity. 

## 3. Results

Symptoms of exacerbation among group I (*n* = 20) were cough, fever, and dyspnea on exertion in 100% of patients, increased sputum 95%, and dyspnea at rest 85%. Laboratory investigations showed that total leucocytic count mean values were 14.76 ± 1.179 cells (×1000/cc). Erythrocyte sedimentation rate in the first-hour (ESR) mean values were 15.85  ±  7.177 (mm/hr). C-reactive protein (CRP) mean values were 23.40  ±  10.465 (mg/L). The mean value of FEV1% was 66.070 ± 10.265. 

In contrast to group II (*n* = 20), total leucocytic count mean values were 9.55 ± 1.22 cells (×1000/cc). Erythrocyte sedimentation rate first-hour (ESR) mean values were 5.47 ± 1.17 (mm/hr). The mean values of FEV1% were 81.435 ± 5.978. These results were statistically significant as increase in mean values of group I when compared to group II. The mean level of exhaled CO among the three studied groups (I, II, and III) was (5.35, 2.65, and 1.3, resp.). This showed a highly statistically significant difference among the three studied groups (*P* < 0.05) Table 1. 

Correlation coefficient test showed a statistically significant negative correlation between eCO levels and pulmonary function data (FEV1,* FVC, FEV1/FVC ratio*)* in both group I and group* II*.*

The severity score done in patients with exacerbation (group I) according to Kanga et al. [8] was negatively correlated with FEV1% and FVC% and positively correlated with the level of exhaled CO in patients of group I which agreed with Kanga et al. [8].

ROC curve showed that the cutoff value of exhaled Co which discriminates between cases and control is 1.5 (ppm) with sensitivity of 100% and specificity of 70% and the cutoff value which discriminates between group I (exacerbation group) and group II (quiescent group) is 3 (ppm) with sensitivity of 100% and specificity of 100%. 

## 4. Discussion

Chronic lung disease (CLD) is a major cause of morbidity and mortality and represents substantial economic and social burden throughout the world [9]. Neutrophilic inflammation and infection lead to injury and breakdown of airway matrix constituents [10]. Inflammation is triggered by inflammatory events as infection or hypersensitivity. Pulmonary exacerbations are a major cause of morbidity and decreased quality of life for patients with chronic lung diseases (CLD) [3]. In the last decade, there has been an increased application of exhaled breath analysis, either considering exhaled gases or exhaled condensates. Exhaled breath analysis has enormous potential as an easy, non invasive means of monitoring inflammation and oxidative stress in the airway [4]. CO represent a product of endogenous metabolic processes whose presence on exhaled breath may fluctuate with systemic, pulmonary, or airway inflammation, and therefore have been proposed as indices of disease states involving inflammatory processes [5]. 

Patients' age (*n* = 40) ranged between 5 to 17 years with a mean age 9.98 ± 3.29 years and this was needed for patients cooperation in performance of pulmonary function testing and CO analysis test which agreed with Freire et al. (2008) [11].

A statistically significant decrease was found in mean values of weight centile for age and sex and height centile for age and sex in patients of group I and patients of group II when compared to control group. This was explained by increased metabolic demand due to chronic illness and chronic lung infection and agreed with Alves et al. (2007) and Rabin et al. (2004) [12, 13].

A statistically significant increase was found in percentage of patients with expectoration, dyspnea at rest, dyspnea on exertion, and fever in patients of group I compared to group II. Also a statistically significant increase was found in percentage of ronchi (sibilant, sonorous), crepitations (fine, coarse), diminished air entry, and respiratory rate (normal rate, tachypnea) in patients of group I when compared to group II. This came in agreement with the criteria of pulmonary exacerbations by Kapur et al. (2012) [14]. 

In agreement with Kapur et al. (2012), Eichler et al. (1999), Tanrikulu et al. (2010), and El-Seify et al. (2011) [14–17], a statistically significant increase in mean values of CRP, ESR, and TLC in patients of group I when compared to group II were found. 

Regarding pulmonary function tests results, patients of group I and group II had a statistically significant lower FEV1% and FVC% mean values when compared to control group which came in accordance to Sanders et al. (2011) and Kapur et al. (2012) [14, 18]. 

 Statistically significant higher mean values of exhaled CO were found in patients of group I followed by patients of group II then control group (5.35, 2.65, 1.3 ppm, resp.). These data agreed with Antuni et al. (2000) and Babusikova et al. (2008) [6, 19].

Statistically significant negative correlation was found between eCO level and pulmonary function data (FEV1% and FVC %) in patients of group I and group II. This came in accordance with Antuni et al. (2000) and Babusikova et al. (2008) [6, 19]. 

The severity score done in patients with exacerbation according to Kanga et al. (1999) [8] was negatively correlated to FEV1%, FVC%. These results agreed with Kanga et al. (1999). Also, the severity score was positively correlated with the level of exhaled CO in patients of group I.

The cutoff value of exhaled CO which discriminates between cases and control is 1.5 (ppm) with sensitivity of 100% and specificity of 70% and the cutoff value which discriminates between group I (exacerbation group) and group II (quiescent group) is 3 (ppm) with sensitivity of 100% and specificity of 100%. 

These findings suggested that increased eCO level may be an indicator of acute exacerbations in children with CLD and can be used as a simple method for monitoring disease progression. 

## 5. Conclusion

Exhaled CO analysis can be considered as a non invasive marker of inflammation for early detection of acute exacerbation of chronic lung diseases. Further studies about exhaled carbon monoxide as a prognostic marker to assess exacerbation and the degree of inflammation in patients with chronic lung diseases are required for deciding the plan of the treatment. 

## Figures and Tables

**Table 1:** Comparison between studied groups regarding exhaled CO level.

	Group I Mean ± SD	Group II Mean ± SD	Group III Mean ± SD	*P* value	Significance
Exhaled CO (ppm)	5.35 ± 1.35	2.65 ± 0.49	1.30 ± 0.47	0.00	HS

HS: highly significant.
